# Multi-Target Tracking AA Fusion Method for Asynchronous Multi-Sensor Networks

**DOI:** 10.3390/s23218751

**Published:** 2023-10-27

**Authors:** Kuiwu Wang, Qin Zhang, Guimei Zheng, Xiaolong Hu

**Affiliations:** 1 School of Air Defense and Missile Defense, Air Force Engineering University, Xi’an 710051, China; wkw19971997@163.com (K.W.); kinzh@263.net (Q.Z.);; 2 Graduate School of Air Force Engineering University, Xi’an 710051, China

**Keywords:** multi-sensor network, arithmetic average fusion, asynchronous multi-target tracking, random finite set, PHD filter

## Abstract

Aiming at the problem of asynchronous multi-target tracking, this paper studies the AA fusion optimization problem of multi-sensor networks. Firstly, each sensor node runs a PHD filter, and the measurement information obtained from different sensor nodes in the fusion interval is flood communicated into composite measurement information. The Gaussian component representing the same target is associated with a subset by distance correlation. Then, the Bayesian Cramér–Rao Lower Bound of the asynchronous multi-target-tracking error, including radar node selection, is derived by combining the composite measurement information representing the same target. On this basis, a multi-sensor-network-optimization model for asynchronous multi-target tracking is established. That is, to minimize the asynchronous multi-target-tracking error as the optimization objective, the adaptive optimization design of the selection method of the sensor nodes in the sensor network is carried out, and the sequential quadratic programming (SQP) algorithm is used to select the most suitable sensor nodes for the AA fusion of the Gaussian components representing the same target. The simulation results show that compared with the existing algorithms, the proposed algorithm can effectively improve the asynchronous multi-target-tracking accuracy of multi-sensor networks.

## 1. Introduction

Nowadays, with the rapid development of information technology, the multi-target scene of sensor monitoring tends to be complicated, and a single sensor can no longer meet the needs of the real environment. To solve the multi-target-tracking problem in complex scenes, one can apply a multi-sensor network system to a multi-target-tracking environment. A multi-sensor multi-target-tracking network system is a multi-sensor information-fusion system that can effectively track multiple targets through information fusion. As a key research direction in the field of multi-target tracking, multi-sensor information-fusion technology has been applied in military and industry [1,2,3,4,5,6,7,8,9].

Distributed multi-sensor networks [10,11] and centralized multi-sensor networks [12] are mainstream multi-sensor fusion architectures. Multi-sensor network systems are used more in multi-target tracking. For multi-sensor multi-target-tracking problems, how to design a reasonable and effective filtering scheme is the key. At present, the two main processing methods for multi-target-tracking problems are traditional multi-target-tracking methods and multi-target-tracking methods based on a random finite set. The former method transforms the multi-target-tracking problem into multiple single-target-tracking problems by using the Bayesian filtering method through data association. The random finite set multi-target-tracking method regards all target states and measurements as random finite sets and then uses multi-target Bayesian filtering technology to simultaneously estimate the number and state of the targets. Compared with the traditional data-association multi-target-tracking method, the random finite set multi-target-tracking method provides a unified, top-down probability theory method for multi-target tracking and does not require an explicit data-association process, and the algorithm complexity is greatly reduced. Based on the theory of the random finite set (RFS), Mahler and Vo et al. proposed a series of multi-target-tracking filters, such as the probability hypothesis density (PHD) filter [13], cardinalized PHD (CPHD) filter [14], multi-target multi-Bernoulli filters [15,16,17,18], etc. A random finite set is a random variable whose value is an unordered finite set; that is, the number and distribution of random variables in a random finite set are random. Intuitively, a random finite set is a random spatial point pattern. They are now widely used in robotics [19], computer vision [20], autonomous driving [21], and traffic monitoring [22]. The continuous development and improvement in random finite set theory and methods have also promoted the development of multi-sensor network systems. Reasonable communication fusion methods can effectively improve the accuracy of multi-target tracking. The fusion method based on the average information consensus [23] has shown good performance for multi-sensor networks. The information flooding proposed by Li Tiancheng [24] avoids the reuse of information and greatly improves the communication efficiency of the network. The main average consensus fusion methods are arithmetic average (AA) fusion and geometric average (GA) fusion. Among them, the fusion based on the generalized covariance intersection (GCI) [25,26,27] is a GA fusion method. The GA fusion method is prone to the problem of repeated calculation, and the fusion performance will decrease when the local sensor fails [28]. The AA fusion method [29,30] can effectively carry out robust and approximately suboptimal distributed fusion, which can effectively deal with any correlation between different sources and has a strong ability to resist local faults and high-frequency missed detection. In recent years, it has attracted the attention of the international academic community [31]. The multi-target density fusion algorithm based on a random finite set has been applied to the problems of heterogeneous multi-sensor multi-target tracking [32], limited field of view multi-sensor fusion [33], and mobile sensor self-localization [34] in practical application scenarios. This method is of great significance for research on actual multi-target tracking.

However, in the actual processing process, the time reference, initial sampling time, and sampling interval of each sensor node may be different due to the constraints of the sensor’s own performance and other conditions. Moreover, due to the network bandwidth and other reasons, the sensor network may have communication delays and other phenomena, which will lead to the target measurement information obtained by the sensor network not being synchronous; that is, the asynchronous problem. Around the problem of asynchronous target tracking, domestic and foreign scholars have conducted a lot of research [35,36]. According to Qiu et al., for asynchronous multi-sensor systems, the basic idea of a sequential filtering algorithm based on the sampling measurement sequence, the asynchronous sampling measurement for left synchronous lifting, proved that the fusion-estimation algorithm based on left and right synchronous lifting technology is equivalent in accuracy [37]. Yu et al. proposed a recursive arithmetic averaging method on time to reduce the communication delay for multi-sensor fusion systems with different sampling rates. They proposed an extended method of multi-sensor CPHD filters to adapt to the environment of an unknown clutter rate and unknown detection probability [38]. The multi-sensor network structure based on the PHD filter [39,40,41] has been proposed and used for asynchronous sensors with different sampling rates. Reference [42] studied a robust PHD fusion algorithm, which can jointly estimate the target state and time offset between sensors. However, using a sampling-based optimization strategy to estimate the time offset at the sensor imposes huge communication and computational requirements.

Aiming at the problem of asynchronous target tracking, a power-allocation algorithm for a multi-static radar system is proposed in [43]. According to the optimal order fusion of measurement information, the expression of the target-tracking accuracy is derived and used as the optimization objective function. Taking the upper and lower limits of the radiation power and the total radiation power limit as constraints, the asynchronous observation model of the multi-static radar system is established, which is solved by the convex relaxation method and gradient projection method. Reference [44] studied a joint-power bandwidth-allocation algorithm for asynchronous radar network target tracking in a cluttered environment. Under the constraints of the given system radiation power and transmission bandwidth constraints, the branch-reduction definition algorithm is used to solve the problem. The simulation results show that the algorithm can significantly improve the target-tracking accuracy compared with the uniform-power bandwidth-allocation comparison algorithm. In [45], two optimal resource-allocation methods are proposed by combining heterogeneous radar networks with asynchronous multi-target tracking, and the radiation power and dwell time of the heterogeneous radar networks in each fusion interval are optimized by combining the gradient projection method and double rise method. In different cases, the system’s multi-target-tracking accuracy and RF stealth performance are effectively improved. Aiming at the multi-target-tracking background of a radar system, Zhang Yongping [46] proposed a resource-management algorithm under a non-ideal detection environment. The Posterior Cramér–Rao Lower Bound (PCRLB) of the target under non-ideal detection was obtained by an enumeration calculation and used as the optimization objective function to optimize the parameters such as the transmitting beam and power to improve the multi-target-tracking accuracy.

The above research results have laid a solid foundation for improving the multi-target-tracking performance of sensor networks. However, in the actual environment, the detection probability of the sensor is less than one due to factors such as the target radar cross section and signal radiation, and the sensor network system cannot always guarantee that all sensor nodes have the same sampling interval. Existing studies have not considered the optimization of the tracking performance for asynchronous multi-target-tracking scenarios in centralized multi-sensor networks in non-ideal environments. Therefore, this paper focuses on the non-ideal detection environment, asynchronous multi-target tracking, and centralized multi-sensor AA fusion theory and studies the centralized multi-sensor AA fusion problem for asynchronous multi-target tracking in a non-ideal detection environment. The problem can better adapt to sensor network systems with different sampling intervals. Firstly, for multi-sensor networks, the measurement information of each sensor node representing the same target is integrated into composite measurement information through distance correlation. Then, for the asynchronous multi-target-tracking problem, an asynchronous multi-target-tracking-optimization model is established to minimize the asynchronous multi-target-tracking error. As the optimization goal, the SQP algorithm is used to optimize the selection of sensor nodes in the sensor network, improving the computational efficiency and the asynchronous multi-target-tracking accuracy of the multi-sensor network. The simulation results show that the proposed algorithm can effectively reduce the asynchronous multi-target-tracking error of multi-sensor networks and improve the tracking accuracy compared with the existing algorithms.

## 2. Problem Background and Model

**Figure 1:**
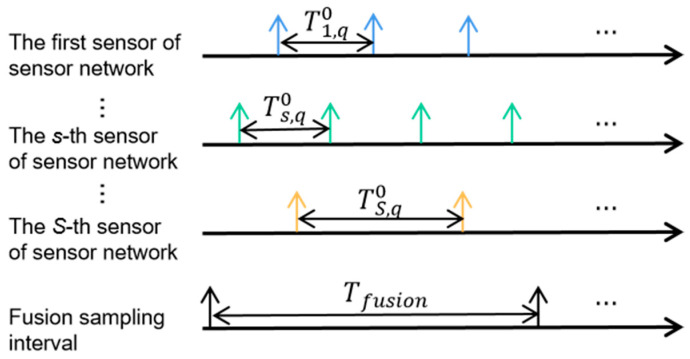
Asynchronous measurement model.

Suppose the multi-sensor network consists of *S* radar nodes, and the coordinate of the *s*-th $\left(s=1,2,\cdots ,S\right)$ radar node in the multi-sensor network is $\left(x_{s},y_{s}\right)$. Due to the different working modes of the sensor nodes in the sensor network, their initial sampling time $t_{s,q}^{ini}$ and sampling interval $T_{s,q}$ may also be different for the target *q*. Based on this, Figure 1 shows the asynchronous measurement model of target *q* in the fusion interval $T_{fusion}$.

In addition, it is assumed that there are *Q* moving targets in the monitoring area of the multi-sensor network system, and the initial position of the target *q* $\left(q=1,2,\cdots ,Q\right)$ is $\left(x_{ini}^{q},y_{ini}^{q}\right)$ and the moving speed is $\left(v_{x,ini}^{q},v_{y,ini}^{q}\right)$.

### 2.1. Random Finite Set Theory

Let X be a random variable; the multi-target state set can be reduced to a finite set $X=\left\{x_{1},\cdots ,x_{n}\right\}\subseteq X$, n≥0. n=0 represents the empty set. The probability density is given a random finite set variable; then, the symmetric joint distribution $p_{n}\left(x_{1},\cdots ,x_{n}\right)$ and the cardinality distribution $\rho \left(n\right)$ can be used to uniquely represent the probability density of the random finite set variable X [15]. The symmetric joint distribution describes the distribution of the elements of the random finite set in the state space, and the potential distribution describes the distribution of the number of points:

(1) $$p\left(X\right)=p\left(\left\{x_{1},\cdots ,x_{n}\right\}\right)=n!\rho \left(n\right)p_{n}\left(x_{1},\cdots ,x_{n}\right)$$

In the formula, the factorial term n! represents all permutations in the joint distribution, and the integral of the probability density can be defined as

(2) $$\int p\left(X\right)\delta X=p\left(\emptyset \right)+\sum_{n=1}^{\infty }\frac{1}{n!}\int p\left(\left\{x_{1},\cdots ,x_{n}\right\}\right)dx_{1}\cdots dx_{n}$$

This formula can be used to describe the multi-objective state space distribution and potential distribution. The integral of $p\left(X\right)$ is 1, namely

(3) $$\begin{array}{l} \int p\left(X\right)\delta X=\rho \left(0\right)+\sum_{n=1}^{\infty }\frac{1}{n!}n!\underset{Standard\;probability\;density\;function=1}{\underset{︸}{\int p_{n}\left(\left\{x_{1},\cdots ,x_{n}\right\}\right)dx_{1}\cdots dx_{n}}}\\ =\sum_{n=0}^{\infty }\rho \left(n\right)=1 \end{array}$$

$\int p\left(X\right)\delta X=1$ shows that $p\left(X\right)$ is a probability density function. The cardinality distribution $\rho \left(n\right)$ is calculated as follows:

(4) $$\rho \left(n\right)=\frac{1}{n!}\int p\left(\left\{x_{1},\cdots ,x_{n}\right\}\right)dx_{1}\cdots dx_{n}$$

The cardinality distribution represents the distribution of the number of multiple targets. The strength function is the first-order statistical moment approximation of the multi-target density. It is a function defined in the single-objective state space. The strength function of a random finite set X can be defined as

(5) $$D\left(x\right)=\int \sum_{x^{'}\in X}\delta_{x^{'}}\left(x\right)p\left(X\right)\delta \left(X\right)$$

In the formula, the $\delta_{x^{\prime }}\left(x\right)$ function is the set-valued Dirac function. Since the integral under the framework of the random finite set is the set-valued integral, the set-valued integral is usually impossible to solve, so the $\delta_{x^{\prime }}\left(x\right)$ function is needed to obtain the approximate value. The $\delta_{x^{\prime }}\left(x\right)$ function is given by the following formula:

(6) $$\delta_{x^{\prime }}\left(x\right)≜\left\{\begin{array}{l} 1,\;\;\;\;if\;x^{\prime }=x\\ 0,\;\;\;\;\;\;\;\;else \end{array}\right.$$

### 2.2. Multi-Target Bayesian Filtering

In the multi-target Bayesian filter, the multi-target state and measurement sets are modeled as random finite sets. Suppose that at time *k*, the multi-target state set $X_{k}\subset X$ and the multi-target measurement set $Z_{k}\subset \mathbb{Z}$ are given. Since $X_{k}$ and $Z_{k}$ are random finite sets, not only will the target state evolve, but the number of targets will change over time also. A random finite set is a finite set of elements that are different, disordered, and variable in number. It can naturally characterize false alarms, missed detection, target birth, and death in multi-target processes.

At time *k*, the multi-target state set can be modeled as

(7) $$X_{k}=S_{k}\left(X_{k-1}\right)\cup B_{k}$$

Among them, $S_{k}$ is the RFS of the surviving target with a survival probability of $P_{s}\left(\cdot \right)$. Each target state is either transferred to a new target state with a survival probability or disappears with a probability of $1-P_{s}\left(\cdot \right)$.When the target disappears, $S_{k}$ is an empty set. In addition, new targets may appear at time *k*, $B_{k}$ is the RFS of the new target, and the items are independent of each other, and the multi-target measurement set can be modeled as

(8) $$Z_{k}=H_{k}\left(X_{k}\right)\cup K_{k}$$

Among them, $H_{k}\left(X_{k}\right)$ is the RFS of the measurements generated by $P_{D}\left(\cdot \right)$, the detection probability is $P_{D}\left(\cdot \right)$, and $K_{k}$ is the RFS of the measurements from clutter. For a given target $x_{k}$, it is either detected with probability $P_{D}\left(\cdot \right)$ or not detected with probability $1-P_{D}\left(\cdot \right)$. When the target is detected, the likelihood function of the measurement $z_{k}$ obtained from $x_{k}$ is $g_{k}\left(z_{k}|x_{k}\right)$. Therefore, at time *k*, each state $x_{k}$ generates a random finite set. When the target is detected, the random finite set is $\left\{z_{k}\right\}$, and when it is not detected, the random finite set is empty.

The following equations give the multi-target Bayesian filter prediction and update equations [15]:

(9) $$\pi_{k|k-1}\left(X_{k}|Z_{k-1}\right)=\int f_{k|k-1}\left(X_{k}|X\right)\pi_{k-1}\left(X|Z_{1:k-1}\right)\delta X$$

(10) $$\pi_{k}\left(X_{k}|Z_{1:k}\right)=\frac{g_{k}\left(Z_{k}|X_{k}\right)\pi_{k|k-1}\left(X_{k}|Z_{1:k-1}\right)}{\int g_{k}\left(Z_{k}|X\right)\pi_{k|k-1}\left(X|Z_{1:k-1}\right)\delta X}$$

where $\pi_{k|k-1}\left(X_{k}|Z_{k-1}\right)$ and $\pi_{k}\left(X_{k}|Z_{1:k}\right)$ are the multi-target prior probability density and posterior probability density, respectively; $g_{k}\left(\cdot |\cdot \right)$ is a multi-target likelihood function; and $f_{k|k-1}\left(\cdot |\cdot \right)$ is the multi-target Markov transition density.

The multi-target Bayesian filter extrapolation is similar to the single-target Bayesian filter extrapolation. The main difference is that the integral in the multi-target Bayesian filter recursion is a set-valued integral while the integral in the single-target Bayesian filter recursion is a variable integral. Since the set-valued integral is usually impossible to solve, it is necessary to find a suboptimal method to approximate the multi-objective Bayesian filter in practical applications.

### 2.3. Single-Sensor Multi-Target-Tracking Method

The PHD filter is run on the sensor node, the target state set is $X_{k}=\left\{x_{k}^{1},\cdots ,x_{k}^{N_{k}}\right\}$, and the measurement set is $Z_{k}=\left\{z_{k}^{1},\cdots ,z_{k}^{M_{k}}\right\}$, where $x_{k}^{n}$ and $z_{k}^{m}$ represent the *n*-th target state and the *m*-th measurement at time k, respectively. $N_{k}$ and $M_{k}$ are the number of targets and measurements at time *k*, respectively. Under the assumption that the prior probability of the multi-target approximately obeys a Poisson distribution, the PHD recursive formula is [13] based on the theory of random finite set statistics:

(11) $$\begin{array}{ll} D_{k|k-1}\left(x\right)= & \int \left(P_{s,k|k-1}f_{k|k-1}\left(x|\zeta \right)\right)+\beta_{k|k-1}\left(x|\zeta \right)D_{k-1|k-1}\left(\zeta \right)d\zeta \\ & +\gamma_{k}\left(x\right)\; \end{array}$$

(12) $$\begin{array}{l} D_{k|k}\left(x\right)=\left[1-P_{D,k}\right]D_{k|k-1}\left(x\right)\\ +\sum_{z_{k}\in Z_{k}}\frac{P_{D,k}g_{k}\left(z|x\right)D_{k|k-1}\left(x\right)}{\lambda c\left(z_{k}\right)+\int P_{D,k}g_{k}\left(z|\zeta \right)D_{k|k-1}\left(\zeta \right)d\zeta } \end{array}$$

$\gamma_{k}\left(x\right)$ and $\beta_{k|k-1}\left(x|\zeta \right)$ represent the RFS intensity function of newborn and derived targets [10]. $P_{s,k|k-1}$ represents the survival probability of the target at time *k* − 1, $P_{D,k}$ represents the detection probability of the target at time *k*, $f_{k|k-1}\left(x|\zeta \right)$ represents the state transition probability density function, $g_{k}\left(z|x\right)$ represents the single-target likelihood function, λ is the average clutter number, and $c\left(z_{k}\right)$ is the clutter probability density function that obeys the Poisson distribution. The multi-target density $\pi \left(X\right)$ of Poisson RFS X is in the following form:

(13) $$\pi \left(X\right)=\exp\left(-\int_{X}D\left(x\right)dx\right)\prod_{x\in X}D\left(x\right)$$

Given a region χ∈X, the number of predicted targets in this region can be calculated as $\int_{x\in \chi }D\left(x\right)dx$, and the total number of predicted targets in the whole state space is $\int_{X}D\left(x\right)dx$.

The PHD filter is based on the following assumptions:

(1) The motion of each target and its measurement are independent of each other.
(2) The random finite set of newborn targets and the random finite set of survival targets are independent of each other.
(3) The clutter random finite set and the measurement generated by the target are independent of each other.

### 2.4. AA Fusion

Assuming that the local fusion density of each sensor node is $p_{i}\left(X\right)$, the corresponding fusion weight is $w_{i}\geq 0$, where i=1,⋯,S and $\sum_{i=1}^{S}w_{i}=1$; then, the definition of AA fusion is as follows:

(14) $$p_{AA}\left(X\right)=\sum_{i=1}^{S}w_{i}p_{i}\left(X\right)$$

The weighted sum of the K-L divergence of the AA fusion results for each local fusion density is the smallest [47,48,49]; that is:

(15) $$p_{AA}\left(X\right)=\underset{g\left(X\right)}{\arg\min}\sum_{i=1}^{S}w_{i}D_{KL}\left(p_{i}\left(X\right)∥g\left(X\right)\right)$$

The K-L divergence of $g\left(X\right)$ with respect to $p\left(X\right)$ is as follows:

(16) $$D_{KL}\left(p\left(X\right)∥g\left(X\right)\right)=\int_{\chi }p\left(X\right)\mathrm{lg}\frac{p\left(X\right)}{g\left(X\right)}\delta X$$

Replacing the above K-L divergence with the Euclidean square distance still holds [50], which means that the AA fusion approximates the minimum entropy of different information sources, which retains all the information of the different information sources.

### 2.5. Target-Motion Model

Considering a discrete-time linear stochastic system with missing measurements from *S* sensors, the loss in measurements occurs randomly. The following systems are considered:

(17) $$x_{k}=F_{k-1}x_{k-1}+w_{k-1},\;k\geq 1$$

(18) $$y_{k}^{s}=\theta_{k}^{s}h_{k}^{s}\left(x_{k}\right)+v_{k}^{s},\;k\geq 1,\;s=1,2,\cdots ,S$$

Among them, $x_{k}$ is the target-state value, $y_{k}^{s}$ is the measurement value collected by the sensor *s* at time *k*, $w_{k-1}$ is the Gaussian white noise with a mean value of 0 and covariance of *Q*, $v_{k}^{s}$ is the measurement noise with a mean value of 0 and covariance of *R*, $F_{k-1}$ is the state-transition matrix of the target, and $h_{k}^{s}$ is the measurement matrix of the sensor *s*. On this basis, a binary variable is defined to describe the pairing of sensor nodes with the target at k time in multi-sensor networks:

(19) $$d_{s,k}^{q}=\left\{\begin{array}{l} 1,\;\;\;\;\;\;the\;target\;q\;is\;detected\;by\;sensor\;s\\ 0,\;\;\;\;\;\;else \end{array}\right.$$

## 3. Asynchronous Multi-Target-Tracking-Optimization Algorithm for Multi-Sensor Networks

### 3.1. Gaussian Mixture Method for Distance-Correlated PHD

The GM-PHD filter expresses the multi-target prior PHD and posterior PHD at *k* − 1 time as a Gaussian mixture $\left\{\omega_{k-1}^{i},m_{k-1}^{i},p_{k-1}^{i}\right\}$, and the Gaussian component of the sensor *s* can be written as

(20) $$D_{s,k-1}\left(x\right)=\sum_{i=1}^{J_{k-1}}\omega_{k-1}^{i}N\left(x;m_{k-1}^{i},p_{k-1}^{i}\right)$$

where $J_{k-1}$ represents the number of Gaussian components at time *k* − 1. $\omega_{k-1}^{i}$ is the weight of the *i*-th Gaussian component and $m_{k-1}^{i}$ and $p_{k-1}^{i}$ are the mean and covariance of the *i*-th Gaussian component, respectively.

Through the information sharing between adjacent sensors, each sensor contains the posterior information of adjacent sensors. The pan-red communication in Reference [24] can effectively and quickly complete the information sharing. Let the number of communication iterations between the sensors be t=0,1,⋯,T, and $S_{s}\left(t\right)$ represents the set of adjacent sensors with a distance of t from the *s*-th sensor. After *t* iterations of the sensor, the posterior probability density set on the sensor is

(21) $$\pi_{s,k}^{\left(t\right)}\left(X\right)=\underset{j\in S_{s}\left(\leq t\right)}{\cup }\pi_{j,k}\left(X_{j}\right)$$

When t=0, $\pi_{s,k}^{\left(0\right)}\left(X\right)=\pi_{s,k}\left(X\right)$.

When described by the Gaussian component $D\left(x\right)$, it can be expressed as follows after *t* iterations:

(22) $${\left\{m_{s,k}^{l},p_{s,k}^{l}\right\}}_{l=1}^{N_{s,k}}=\underset{j\in S_{s}\left(\leq t\right)}{\cup }{\left(m_{j,k}^{l},p_{j,k}^{l}\right)}_{l=1}^{N_{j,k}}$$

where

(23) $$N_{s,k}\left(t\right)=N_{s,k}+\sum_{j\in S_{s}\left(\leq t\right)}N_{j,k}$$

After the communication is completed, each Gaussian component represents different targets, so direct fusion cannot obtain better results. In this paper, the Gaussian component after communication is correlated with distance, and the Gaussian component of the same target is correlated with the same subset. The fusion of the Gaussian component subset after the distance correlation can reduce the adverse effects of clutter, filter out false targets, and greatly improve the accuracy of multi-target tracking.

The correlation of Gaussian components uses the distance-correlation method [38] to calculate the distance between the Gaussian mean $m_{s,k}^{i}$ and $m_{s',k}^{j}$ of the Gaussian components from different sensors $s'\in S\left(\leq t\right)$ after communication on the sensor *s*:

(24) $$d={\left(m_{s,k}^{i}-m_{s',k}^{j}\right)}^{T}Q_{s,k}^{-1}\left(m_{s,k}^{i}-m_{s',k}^{j}\right)$$

Here, $Q_{s,k}$ is the process noise covariance matrix, and the threshold $D_{\max}$ is set to control the correlation subset. If the distance $d<D_{\max}$ between the two Gaussian components is considered the same target, the two Gaussian components can be put into a subgroup.

### 3.2. BCRLB Derivation of Asynchronous Sensor Networks in Non-Ideal Detection Environment

At the fusion time *k*, combined with the pairing index $\theta_{k}^{s,q}$ of the sensor node *s* and the target *q*, the measurement equation of the sensor node *s* to the target *q* can be obtained:

(25) $$z_{k}^{s,q}=\left\{\begin{array}{l} h_{k}^{s}\left(x_{k}^{q}\right)+v_{k}^{s},\;if\theta_{k}^{s,q}=1\\ 0,\;\;\;\;\;if\theta_{k}^{s,q}=0 \end{array}\right.$$

The calculation formula of $H_{k}^{s}\left(x_{k}^{q}\right)$ can be written as follows:

(26) $$H_{k}^{s}\left(x_{k}^{q}\right)=\left[\begin{array}{l} \sqrt{{\left(p_{x,k}-p_{x,k}^{r,q}\right)}^{2}+{\left(p_{y,k}-p_{y,k}^{r,q}\right)}^{2}}\\ \arctan\left(\left(p_{y,k}-y_{s}\right)/\left(p_{x,k}-x_{s}\right)\right) \end{array}\right]$$

$p_{x,k}^{r,q}$ and $p_{y,k}^{r,q}$ represent the actual position of the target *q*; $x_{s}$ and $y_{s}$ represent the absolute position of the sensor; and the covariance matrix of $v_{k}^{s}$ is $R_{k}^{s}=diag\left\{\sigma_{k,r}^{2},\sigma_{k,\theta }^{2}\right\}$, where $\sigma_{k,r}$ represents the distance error and $\sigma_{k,\theta }$ represents the azimuth error.

The relationship between covariance and sensor-related emission parameters can be expressed as [51]:

(27) $$\left\{\begin{array}{l} \sigma_{k,r}^{2}\propto {\left\{P_{k}^{s,q},T_{k}^{s,q}{\left[\beta_{k}^{s,q}\right]}^{2}\right\}}^{-1}\\ \sigma_{k,\theta }^{2}\propto {\left\{P_{k}^{s,q},T_{k}^{s,q}/B_{NN}\right\}}^{-1} \end{array}\right.$$

where $P_{k}^{s,q}$ represents the radiation power of the sensor *s* to the target *q*; $T_{k}^{s,q}$ represents the dwell time of sensor *s* to target *q*; $\beta_{k}^{s,q}$ represents the bandwidth of the transmitted signal; and $B_{NN}$ represents the beam width of the receiving antenna. It can be seen that the radiation power and dwell time of the sensor node are related to the measurement error of the target.

Since the Bayesian Cramér–Rao Lower Bound (BCRLB) can provide a lower bound for the Mean Square Error (MSE) of parameter unbiased estimation [51], this index can be used as a measure of the target-tracking accuracy. At the fusion time *k*, the distance correlation is performed according to Formula (24) to obtain the composite measurement information of different sensor nodes about the target *q*, which is listed in order:

(28) $$M_{k}^{q}={\left[m_{1,k}^{q,1},\cdots ,m_{s,k}^{q,S_{s,k}^{q}},\cdots m_{S,k}^{q,S_{S,k}^{q}}\right]}^{T}$$

where *S* is the number of sensor nodes in the sensor network and $S_{S,k}^{q}$ is the number of Gaussian components of the target q after the distance correlation of the sensor s.

The target BCRLB is derived by combining the composite measurement information. Under ideal detection, at the fusion time *k*, the BCRLB expression of the target *q* state estimation error can be written as [52]

(29) $$C_{BCRLB,k}^{q}={\left[\begin{array}{l} {\left[Q_{k-1}^{q}+F^{q}J^{-1}\left(x_{k-1}^{q}\right){\left(F^{q}\right)}^{T}\right]}^{-1}\\ +\sum_{s=1}^{S}\theta_{k}^{s,q}{\left(H_{s,k}^{q}\right)}^{T}{\left(R_{k}^{s}\right)}^{-1}H_{s,k}^{q} \end{array}\right]}^{-1}$$

Here, $H_{s,k}^{q}$ is the Jacobian matrix of $h_{k}^{s}$.

However, in the non-ideal detection environment, the sensor nodes in the asynchronous multi-sensor network do not successfully detect all the tracking targets, and there may be missed detection. Therefore, according to this situation, a binary variable is defined to represent the detection of the target *q* by the asynchronous multi-sensor network at *k* time:

(30) $$d_{s,k}^{q}=\left\{\begin{array}{l} 1,\;\;\;the\;target\;q\;is\;detected\;by\;sensor\;s\\ 0,\;\;\;else \end{array}\right.$$

There are *S* sensor nodes in asynchronous multi-sensor networks. Therefore, there are detection situations at each fusion time. These situations are described in detail as

(31) $$G_{k}^{q}=\left\{G_{i,k}^{q}|i=1,2,\cdots ,2^{S}\right\}$$

Here, $G_{i,k}^{q}$ denotes the *i*-th detection of the target *q* by the asynchronous multi-sensor network at the fusion time *k*. Assume that in the *i*-th detection case, there are $\phi_{i,k}^{q}$ sensor nodes in the multi-sensor network that can detect the target *q*; then, the number of radar nodes that do not detect the target q is $S-\phi_{i,k}^{q}$, so the probability of $G_{i,k}^{q}$ is defined as

(32) $$\mathrm{Pr}\left\{G_{i,k}^{q}\right\}={\left(P_{D,s,k}^{q}\right)}^{\phi_{i,k}^{q}}\cdot {\left(1-P_{D,s,k}^{q}\right)}^{S-\phi_{i,k}^{q}}$$

Among them, $P_{D,s,k}^{q}$ represents the detection probability of sensor s. The detection probability of different sensor nodes may be different. In the subsequent calculation, the detection probability is set to a constant, which is convenient for formula derivation and calculation.

According to Formulas (29)–(32), under the non-ideal detection probability, combined with the number of Gaussian components $S_{S,k}^{q}$ of each sensor to the target *q*, the asynchronous tracking error BCRLB of the fusion time *k* to the target *q* is calculated as

(33) $${\tilde{C}}_{BCRLB}^{q}=\sum_{i=1}^{2^{S}}J^{-1}\left(x_{k}^{q}|G_{i,k}^{q}\right)\cdot \mathrm{Pr}\left\{G_{i,k}^{q}\right\}$$

$J\left(x_{k}^{q}|G_{i,k}^{q}\right)$ denotes the Bayesian Information Matrix (BIM) of the target state, which can be written as

(34) $$\begin{array}{l} J\left(x_{k}^{q}|G_{i,k}^{q}\right)={\left[Q_{k-1}^{q}+F^{q}J^{-1}\left(x_{k-1}^{q}|G_{i,k-1}^{q}\right){\left(F^{q}\right)}^{T}\right]}^{-1}\\ +\sum_{s=1}^{S}\sum_{j=1}^{S_{s,k}^{q}}\theta_{k}^{s,q}d_{s,k}^{q}{\left(H_{s,k}^{q,j}\right)}^{T}{\left(R_{k}^{s}\right)}^{-1}H_{s,k}^{q,j} \end{array}$$

It can be seen from Formula (34) that $J\left(x_{k}^{q}|G_{i,k}^{q}\right)$ is related to the matching index $\theta_{k}^{s,q}$ of the sensor and the target, the detection index $\theta_{k}^{s,q}$ of the sensor to the target, the number of Gaussian components $S_{s,k}^{q}$ of the sensor against the target at the fusion time *k,* and the covariance matrix $R_{k}^{s}$. Therefore, the information matrix is affected by the selection of sensor nodes at the fusion time *k* and the radiation power and dwell time.

The first element ${\tilde{C}}_{BCRLB,k}^{q}\left(1,1\right)$ and the second element ${\tilde{C}}_{BCRLB,k}^{q}\left(2,2\right)$ on the diagonal of the information matrix can be used to represent the distance lower bound of the MSE, so it can be used as a measure of the accuracy of asynchronous multi-target tracking:

(35) $$F\left(\theta_{k}^{q},P_{k}^{q},T_{k}^{q}\right)≜\sqrt{{\tilde{C}}_{BCRLB,k}^{q}\left(1,1\right)+{\tilde{C}}_{BCRLB,k}^{q}\left(2,2\right)}$$

Here, $\theta_{k}^{q}$, $P_{k}^{q}$, and $T_{k}^{q}$ are the sensor node selection, the radiation power, and the dwell time matrix of the asynchronous multi-sensor network concerning target *q*, respectively:

(36) $$\left\{\begin{array}{l} \theta_{k}^{q}={\left[\theta_{1,k}^{q},\cdots ,\theta_{s,k}^{q},\cdots ,\theta_{S,k}^{q}\right]}^{T}\\ P_{k}^{q}={\left[P_{1,k}^{q},\cdots ,P_{1,k}^{q},\cdots ,P_{S,k}^{q}\right]}^{T}\\ T_{k}^{q}={\left[T_{1,k}^{q},\cdots ,T_{s,k}^{q},\cdots ,T_{S,k}^{q}\right]}^{T} \end{array}\right.$$

### 3.3. Establishment and Solution of Optimization Model

In the non-ideal detection environment, this paper proposes an asynchronous multi-target-tracking-optimization algorithm for multi-sensor networks, focusing on the asynchronous problem of multi-sensor networks in tracking targets. To simplify the subsequent solution, the radiation power and residence time of each sensor node in the sensor network are the same. By optimizing the sensor-node-selection method at each fusion time, the asynchronous multi-target-tracking error of multi-sensor networks is minimized.

When tracking asynchronous multi-targets, optimizing the algorithm at different Gaussian component arrival times of other sensor nodes is unreasonable. Therefore, the asynchronous multi-target-tracking-optimization algorithm is applied to each fusion time. Suppose that each sensor node uses the same parameters to illuminate the target *q*:

(37) $$\left\{\begin{array}{l} P_{1,k}^{q}=\cdots =P_{s,k}^{q}=\cdots =P_{S,k}^{q}\\ T_{1,k}^{q}=\cdots =T_{s,k}^{q}=\cdots =T_{S,k}^{q} \end{array}\right.$$

According to Formula (37), the optimization model can be established by Formula (38).

(38) $$\left\{\begin{array}{l} \underset{\theta_{k}^{q},P_{k}^{q},T_{k}^{q},\forall q}{\min}F\left(\theta_{k}^{q},P_{k}^{q},T_{k}^{q}\right)\\ P_{1,k}^{q}=\cdots =P_{s,k}^{q}=\cdots =P_{S,k}^{q}\\ T_{1,k}^{q}=\cdots =T_{s,k}^{q}=\cdots =T_{S,k}^{q}\\ \sum_{q=1}^{Q}\theta_{s,k}^{q}\leq 1,\forall s\\ \sum_{s=1}^{S}\theta_{s,k}^{q}=L_{\max},\forall q\\ \theta_{s,k}^{q}\in \left\{0,1\right\},\forall s,q \end{array}\right.$$

Among them, $\sum_{q=1}^{Q}\theta_{s,k}^{q}\leq 1,\forall s$ means that at the fusion time *k,* a sensor node can track *Q* targets at the same time, and $\sum_{s=1}^{S}\theta_{s,k}^{q}=L_{\max},\forall q$ means that at the fusion time *k,* an asynchronous multi-sensor network can assign $L_{\max}$ sensor nodes to track the targets. Due to the non-ideal detection environment, the fourth constraint represents the number of sensor nodes that track the target.

The optimization algorithm aims to solve the optimal sensor node selection in the asynchronous multi-target-tracking problem. Based on this, the sequential quadratic programming (SQP) algorithm is used to solve the problem. The specific method is to specify the target at the initial fusion time of the multi-sensor network for tracking and set the corresponding sensor-emission parameters for irradiation. At the same time, the binary variable $\theta_{s,k}^{q}\in \left\{0,1\right\}$ is relaxed, and the model can be written as

(39) $$\begin{array}{l} \underset{\theta_{k}^{q},P_{k}^{q},T_{k}^{q},\forall q}{\min}F\left(\theta_{k}^{q},P_{k}^{q},T_{k}^{q}\right)\\ \left\{\begin{array}{l} \sum_{q=1}^{Q}\theta_{s,k}^{q}\leq 1,\forall s\\ 0\leq \theta_{s,k}^{q}\leq 1,\forall s,q \end{array}\right. \end{array}$$

It can be seen from Formula (39) that the binary variable $\theta_{s,k}^{q}\in \left\{0,1\right\}$ is relaxed to $0\leq \theta_{s,k}^{q}\leq 1$, and the objective function of the optimization model is only related to the constraint condition $\theta_{s,k}^{q}$. The optimization problem is a convex problem. The optimal sensor node selection can be obtained by solving the optimized model. Firstly, the SQP algorithm is used to solve the optimal-selection coefficient matrix ${\bar{\mu }}_{k}^{q}$ of the sensor node, and then the selection coefficients in the coefficient matrix are arranged in descending order. The radar node corresponding to the coefficient is selected according to $L_{\max}$ until the number of sensor nodes meets $L_{\max}$. Finally, the relaxed binary variable of the sensor-chosen node is changed to $\theta_{S,k}^{q}=1$, and the binary variable of the unselected sensor node is set to 0.

After selecting the optimal sensor node for the target *q*, the next tracking target can be specified. Repeat the above steps to know that all the tracking targets are assigned to the optimal sensor node. Finally, the cycle minimum method terminates the process until the asynchronous target-tracking accuracy is less than a predetermined threshold. According to this method, the optimal sensor node selection for asynchronous multi-target tracking in multi-sensor networks can be obtained.

### 3.4. Sensor Node Fusion Weight

When the sensor nodes for each target are selected, it is necessary to perform AA fusion on the Gaussian components of each sensor for the target, so the corresponding sensor fusion weights need to be set. In the description of the previous problem, the binary variable $\theta_{s,k}^{q}\in \left\{0,1\right\}$ is relaxed $0\leq \theta_{s,k}^{q}\leq 1$ and then solved by the SQP algorithm. The obtained selection coefficient $\left(\mu_{1,k}^{q},\cdots ,\mu_{L_{\max},k}^{q}\right)$ satisfies the minimization of the asynchronous multi-target-tracking error. Therefore, the selection coefficient solved by the SQP algorithm can be used as the multi-sensor fusion weight ${\tilde{w}}_{s,k}^{q}$, and ${\tilde{w}}_{s,k}^{q}$ is the normalized weight:

(40) $${\tilde{w}}_{s,k}^{q}=\frac{\mu_{s,k}^{q}}{\sum_{s=1}^{L_{\max}}\mu_{s,k}^{q}}$$

Here, ${\tilde{w}}_{s,k}^{q}$ represents the fusion weight of the selected sensor node *s* in the AA fusion process.

Next, AA fusion is performed on the selected sensor nodes and the distance-associated Gaussian components. It is assumed that the set of sensor nodes where the associated Gaussian components are located is $S_{k}^{\left(c\right)}$. Then, AA fusion is performed on the parameters of each associated subset as follows:

(41) $${\bar{\omega }}_{k}^{q}=\sum_{s\in S_{k}^{\left(c\right)}}{\tilde{w}}_{s,k}^{q}\omega_{s,k}^{q}$$

(42) $${\bar{m}}_{k}^{q}=\sum_{s\in S_{k}^{\left(c\right)}}{\tilde{w}}_{s,k}^{q}m_{s,k}^{q}$$

(43) $${\bar{p}}_{k}^{q}=\sum_{s\in S_{k}^{\left(c\right)}}{\tilde{w}}_{s,k}^{q}\left(p_{s,k}^{q}+\left({\bar{m}}_{k}^{q}-m_{s,k}^{q}\right){\left({\bar{m}}_{k}^{q}-m_{s,k}^{q}\right)}^{T}\right)$$

Therefore, the fused target density can be written as:

(44) $$D_{AA,k}^{q}=\sum_{i\in J_{M}}{\bar{\omega }}_{k}^{q}N\left(x;{\bar{m}}_{k}^{q},{\bar{p}}_{k}^{q}\right)$$

Among them, $J_{M}$ is the index set of the fused Gaussian component.

### 3.5. Algorithm Flow

The asynchronous multi-target-tracking-optimization algorithm for multi-sensor networks is summarized as follows:

Input: the filtered Gaussian component ${\left\{{\left\{\omega_{s,k-1}^{i},m_{s,k-1}^{i},p_{s,k-1}^{i}\right\}}_{i=1}^{M_{s,k-1}}\right\}}_{s=1}^{S}$ at time *k* − 1, the measurement set $Z_{k}^{R}=\left\{Z_{1,k}^{R},Z_{2,k}^{R},\cdots ,Z_{S,k}^{R}\right\}$ at time k.

For each sensor *s*, the GM-PHD filter is run on the basis of $Z_{s,k}^{R}$ to obtain ${\left\{\omega_{s,k}^{i},m_{s,k}^{i},p_{s,k}^{i}\right\}}_{i=1}^{M_{s,k}}$, where $M_{s,k}$ represents the number of Gaussian components.

for s=1,⋯,S

for t=0,1,⋯,T

Then, ${\left\{\omega_{s,k}^{i},m_{s,k}^{i},p_{s,k}^{i}\right\}}_{i=1}^{M_{s,k\left(T\right)}}$ is obtained by communication through Equation (22), and $M_{s,k\left(T\right)}$ represents the Gaussian component on the sensor s after communication.

end

According to Equation (24), the distance correlation is performed to obtain ${\left\{{\left\{\omega_{s,k}^{q,i},m_{s,k}^{q,i},p_{s,k}^{q,i}\right\}}_{i=1}^{M_{s,k}}\right\}}_{q=1}^{Q}$, where *Q* represents the number of subsets representing the same target, and the composite measurement information of different sensor nodes about the target *q* is listed as $M_{k}^{q}={\left[m_{1,k}^{q,1},\cdots ,m_{s,k}^{q,S_{s,k}^{q}},\cdots m_{S,k}^{q,S_{S,k}^{q}}\right]}^{T}$ in order.

for q=1,⋯,Q

According to Formula (37) and (38), the target BCRLB is deduced, the optimization model is established and solved, and the selection coefficient $\left(\mu_{1,k}^{q},\cdots ,\mu_{L_{\max},k}^{q}\right)$ of each sensor node is obtained by the SQP algorithm.

According to the maximum number of sensor nodes $L_{\max}$, the sensor nodes with larger coefficients are selected as the tracking nodes of the target *q*.

Finally, the fusion weight of the sensor nodes is reset according to the selection coefficient, and the tracking information of different sensor nodes on the target *q* is AA fused.

end

Output: according to ${\left\{{\bar{\omega }}_{k}^{q},{\bar{m}}_{k}^{q},{\bar{p}}_{k}^{q}\right\}}_{q=1}^{Q}$, the target state is extracted, and finally $X_{s,k}$ is obtained.

## 4. Simulation Results and Discussion

To verify the feasibility and superiority of the multi-sensor AA fusion method for asynchronous multi-target tracking proposed in this paper, the following simulation scenarios are designed. The sensor network includes eight sensor nodes. Each sensor node runs a GM-PHD filter. The entire scene can be detected by the sensor node. The detection probability of each sensor node *s* is constant; that is, $P_{D,s,k}=0.95$. The radiation power and dwell time of each sensor node are set to $P_{s,k}^{q}=300\;W$ and $T_{s,k}^{q}=0.006\;s$, respectively. Then, the initial sampling time and sampling interval of each sensor node are given in Table 1. The multi-sensor network is set to allocate $L_{\max}=4$ sensor nodes to fuse each piece of target information. To verify the effectiveness of the proposed algorithm in multi-target-tracking scenarios, the improved algorithm is compared with the traditional GCI fusion algorithm and AA fusion algorithm.

The tracking scene is set to multiple targets appearing in four possible locations or derived from other targets, and the observation area is $\left[-1500,1500\right]\times \left[-1000,1000\right]\;\left(m^{2}\right)$. There are seven targets in the scene. For simplicity, it is assumed that each target performs uniform linear motion.

The state vector of the target is composed of position and velocity components, $x_{k}=\left[p_{x,k}\;p_{y,k}{\;v}_{x,k}{\;v}_{y,k}\right]$, and its state equation is

(45) $$x_{k}=\left[\begin{array}{cccc} 1 & 0 & T & 0\\ 0 & 1 & 0 & T\\ 0 & 0 & 1 & 0\\ 0 & 0 & 0 & 1 \end{array}\right]x_{k-1}+\left[\begin{array}{cc} T^{2}/2 & 0\\ 0 & T^{2}/2\\ T & 0\\ & T \end{array}\right]w_{k-1}$$

The fusion interval $T_{fusion}$ is 1 s, the total tracking time is 100 s, and the process noise $w_{k}∼N\left(0,5\right)$. The intensity of the new target is as follows (46):

(46) $$\gamma_{k}\left(x\right)=\sum_{i=1}^{4}\omega_{\gamma ,k}^{i}N\left(x;m_{\gamma ,k}^{i},p_{\gamma ,k}^{i}\right)$$

**Figure 2:**
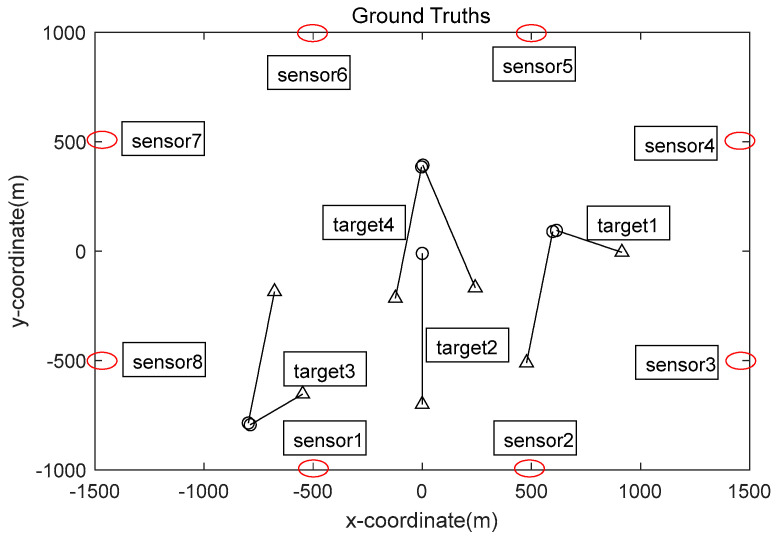
Sensor network node distribution and target trajectory.

Among them, $m_{\gamma ,k}^{1}={\left[0;0;0;-10\right]}^{T}$, $m_{\gamma ,k}^{2}={\left[0;3;400;-7\right]}^{T}$, $m_{\gamma ,k}^{3}={\left[-800;3;-800;15\right]}^{T}$, $m_{\gamma ,k}^{4}={\left[600;15;100;-5\right]}^{T}$, the weight of the newborn target $\omega_{\gamma ,k}^{i}=0.03$, the process noise of the newborn target obeys the Gaussian distribution, the mean value is zero, and the covariance is $Q_{sp,k}^{i}=diag\left(\left[100,100,100,100\right]\right)$. The positions of sensor nodes 1–8 are $\left[-500;0\right]$, $\left[500;0\right]$, $\left[1500;-500\right]$, $\left[1500;500\right]$, $\left[500;1000\right]$, $\left[-500;1000\right]$, $\left[-1500;500\right]$, and $\left[-1500;-500\right]$, respectively. Figure 2 shows the multi-sensor network node distribution and multi-target motion-trajectory diagram.

In the Gaussian component pruning and merging part, the truncation threshold of the Gaussian component is set to $10^{-5}$. The state extraction threshold is set to 0.5, the merging threshold is set to 10, and the maximum number of Gaussian components is 100. The number of Monte Carlo simulations is 100. The tracking quality is evaluated by the OSPA distance:

(47) $$OSPA_{p,c}\left(x_{k},{\hat{x}}_{k}\right)=\sqrt[{p}]{\frac{\min\sum_{i=1}^{\left|x_{k}\right|}{\left(d_{c}\left(x_{k}^{i},{\hat{x}}_{k}^{\pi \left(i\right)}\right)\right)}^{p}+c^{p}\left(\left|{\hat{x}}_{k}\right|-\left|x_{k}\right|\right)}{\left|{\hat{x}}_{k}\right|}}$$

Among them, $x_{k}$ is the target-state vector, and the two parameters of the OSPA distance are set to p=1 and c=200, respectively. The smaller the OSPA distance is, the higher the accuracy of the target-state estimation is.

For the sensor *s*, the measurement vector is the position information, $z_{s,k}=\left[p_{s,z_{x},k}\;p_{s,z_{y},k}\right]$, and the measurement equation is given by the following formula:

(48) $$z_{s,k}=\left[\begin{array}{cccc} 1 & 0 & 0 & 0\\ 0 & 1 & 0 & 0 \end{array}\right]x_{k}+v_{s,k}$$

The measurement noise is $v_{s,k}∼N\left(0,5\right)$. The clutter follows a uniformly distributed Poisson RFS, averaging 60 clutter points per scan $\left(\lambda =60\right)$.

**Figure 3:**
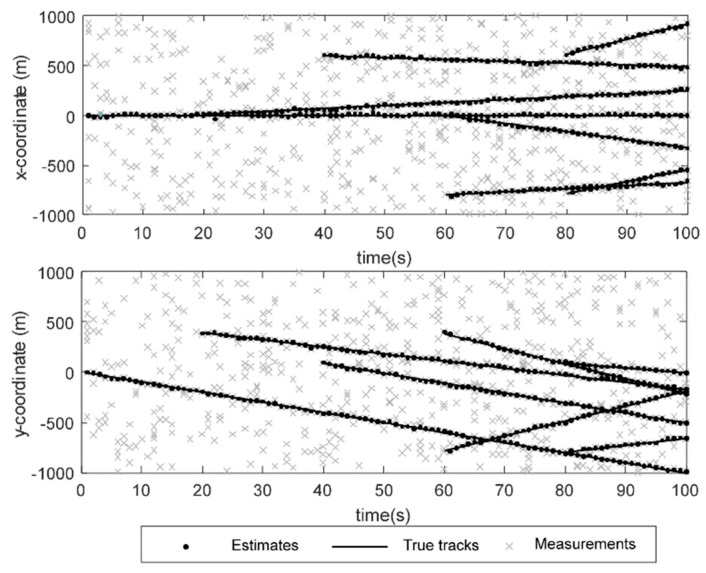
Asynchronous multi-target-tracking results of sensor networks.

Figure 3 shows the asynchronous multi-target-tracking results of the sensor network. Although there will also be individual target-fusion errors and the clutter will be fused as a real target, the multi-sensor network will quickly filter out the error fusion and combine the real target. A better fusion-estimation effect is obtained. From the tracking results of Figure 3, it can be seen that there is no redundant estimation value after the selection of sensor nodes by the sensor network.

**Figure 4:**
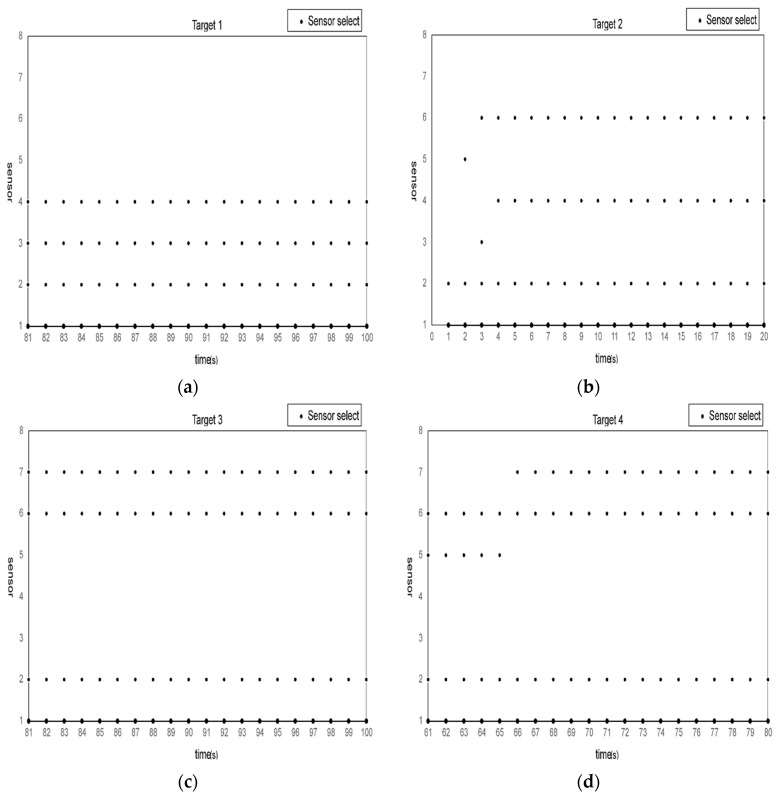
Each target’s radar-node-selection results: (**a**) target 1 sensor-node-selection results; (**b**) target 2 sensor-node-selection results; (**c**) target 3 sensor-node-selection results; (**d**) target 4 sensor-node-selection results.

Figure 4 shows the selection of sensor nodes for each target in the sensor network, where the black dots represent the sensor nodes selected at each fusion time. It can be seen that the multi-sensor network will preferentially select the sensor nodes closer to the target for fusion when selecting the sensor nodes, because when the target is detected by multiple sensor nodes, the Gaussian component weight corresponding to the closer distance is larger, and it is easier to be identified as the real target. Each sensor node will also adaptively optimize the allocation as the target moves, thereby minimizing the asynchronous multi-target-tracking error of the multi-sensor network and improving the multi-target-tracking performance. In addition, the selection of sensor nodes is also directly related to the sampling interval time. For example, for target 1, the sensor network selects sensor node 1, sensor node 2, sensor node 3, and sensor node 4 and does not choose sensor node 5. The reason is that the sampling interval of sensor node 5 is longer, and the sampling included in the fusion time interval of the sensor network is less. Therefore, node 5 is not selected when selecting the sensor nodes for target 1. If the sampling interval is long, it is easy to cause the sensor node to stop tracking of the target. Moreover, the initial sampling time of the sensor nodes will also affect the node selection of the sensor networks for asynchronous multi-target tracking, such as the sensor networks for target 2. The reason is that the number of sampling times in the same fusion interval is less, and the sensor measurements that can be obtained will become less, leading to the increase in asynchronous multi-target-tracking errors.

To effectively prove that the proposed algorithm improves the performance of asynchronous multi-target tracking in multi-sensor networks, this section compares the improved algorithm with the AA fusion and GCI fusion algorithms and analyzes their performance differences.

**Figure 5:**
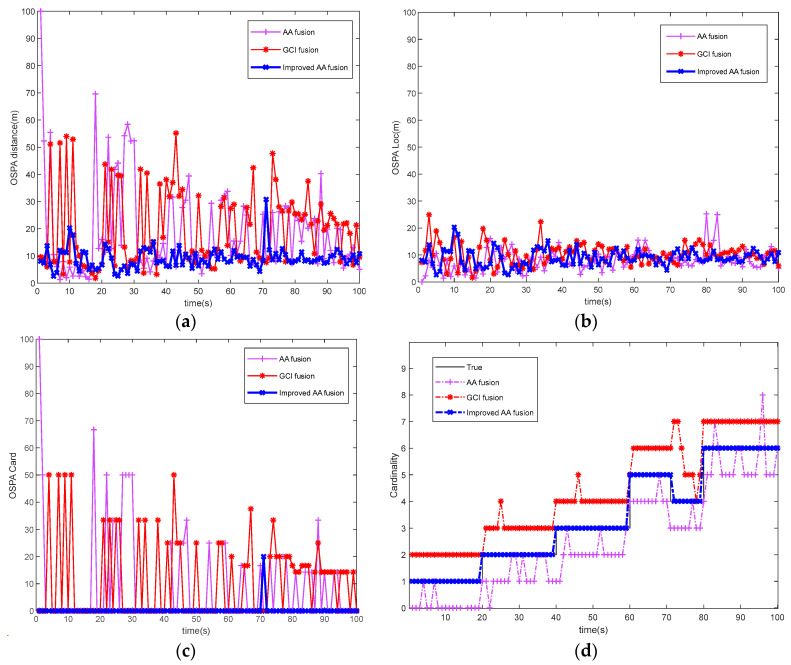
Performance comparison of different algorithms: (**a**) OSPA distance comparison; (**b**) OSPA location comparison; (**c**) target cardinality estimation error; (**d**) multi-target-number estimation.

Figure 5 shows each algorithm’s tracking error comparison map and multi-target-number-estimation comparison map. It can be seen from Figure 5a–c that the tracking error of the improved AA fusion algorithm is significantly lower than that of the other two algorithms. The reason is that the improved AA fusion algorithm adopts the distance-correlation method, significantly reducing clutter’s impact. Then, the selection of sensor nodes is optimized by the optimization model, which increases the robustness of the sensor network. Figure 5d shows the results of each algorithm for multi-target-number estimation. The improved AA fusion algorithm can complete the multi-target-number estimation well. Both the AA fusion algorithm and the GCI fusion algorithm have errors in number estimation. The reason is that the lack of distance-correlation steps does not eliminate the impact of clutter, leading to number-estimation errors in the fusion process. On the other hand, the reason is that the sensor network does not optimize the selection of sensor nodes. An asynchronous multi-sensor network leads to inconsistent sensor node information in the fusion process, which in turn causes number-estimation errors and directly reduces the accuracy of multi-target tracking.

**Figure 6:**
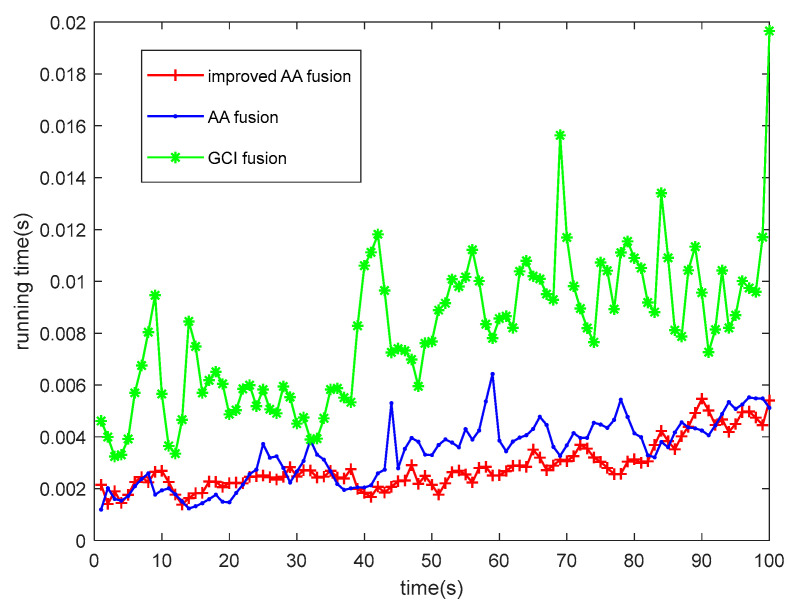
Comparison of running time of each algorithm.

Figure 6 shows the running time comparison of each algorithm. The GCI fusion algorithm has the longest running time because of its algorithm structure. The improved AA fusion algorithm has a particular improvement in the running efficiency compared with the AA fusion algorithm. The reason is that the target measurement information is associated, thereby reducing the calculation of redundancy and ultimately reducing the algorithm’s running time.

**Figure 7:**
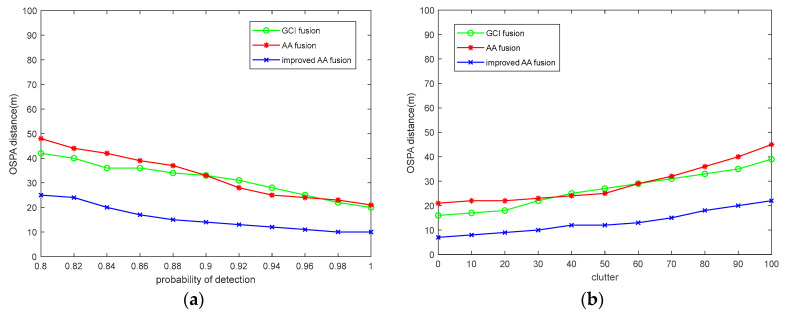
Comparison of OSPA distance of each algorithm under the change in detection probability and clutter rate: (**a**) detection probability change; (**b**) clutter rate change.

To better show the influence of the detection probability and clutter rate changes on the accuracy of asynchronous multi-target tracking in multi-sensor networks, Figure 7 shows the performance comparison of each algorithm under different detection probabilities and different clutter rates. It can be seen from the graph that the improved AA fusion algorithm has the lowest OSPA distance error when the detection probability changes and the clutter rate changes, which also directly proves the robustness of the improved AA fusion algorithm in the asynchronous multi-target-tracking environment of multi-sensor networks.

## 5. Conclusions

This paper considers the problem of asynchronous multi-target tracking in multi-sensor networks under a non-ideal detection environment. It proposes a multi-sensor network AA optimization algorithm for asynchronous multi-target tracking. The optimization algorithm first distinguishes the real target from the clutter by using the distance correlation in the dense clutter environment. Then, it minimizes the asynchronous multi-target-tracking error of the multi-sensor network as the optimization target. Under the premise of satisfying the given radiation power and dwell time, the sensor-node-selection method is adaptively optimized to improve the asynchronous multi-target-tracking accuracy of the multi-sensor network. The simulation results show that the improved algorithm proposed in this paper can effectively reduce multi-sensor networks’ asynchronous multi-target-tracking errors and improve the tracking performance. However, the simulation experiment in this paper is mainly based on linear moving targets. In an actual complex environment, the maneuvering targets are more frequent and the detection probability will change with the change in the combat environment. The next step will focus on multi-sensor-network multi-maneuvering target tracking under the change in the detection probability.

## Figures and Tables

**Table 1:** Initial sampling time and sampling interval of each sensor node.

s	$t_{s,q}^{ini}$	$T_{s,q}$
1	1	0.2
2	1	0.2
3	3	0.4
4	4	0.2
5	2	0.6
6	3	0.4
7	4	0.2
8	3	0.6

## Data Availability

No new data were created or analyzed in this study. Data sharing is not applicable to this article.
